# Physical activity and screen time of children and adolescents before and during the COVID-19 lockdown in Germany: a natural experiment

**DOI:** 10.1038/s41598-020-78438-4

**Published:** 2020-12-11

**Authors:** Steffen C. E. Schmidt, Bastian Anedda, Alexander Burchartz, Ana Eichsteller, Simon Kolb, Carina Nigg, Claudia Niessner, Doris Oriwol, Annette Worth, Alexander Woll

**Affiliations:** 1 Karlsruhe Institute of Technology, Engler-Bunte-Ring 15, 76131 Karlsruhe, Germany; 2 University of Education Karlsruhe, Bismarckstraße 10, 76133 Karlsruhe, Germany

**Keywords:** Health care, Risk factors

## Abstract

The impact of COVID-19 on social life has been drastic and global. However, the different numbers of cases and different actions in different countries have been leading to various interesting yet unexplored effects on human behavior. In the present study, we compare the physical activity and recreational screen time of a representative sample of 1711 4- to 17-year-olds before and during the strictest time of the first COVID-19 lockdown in Germany. We found that sports activity declined whereas recreational screen time increased. However, a substantial increase in habitual physical activities leads to an overall increase in physical activity among children and adolescents in Germany. The effects differ in size but not in their direction between age groups and are stable for boys and girls. We conclude from this natural experiment that physical activity among children and adolescents is highly context-driven and mutual and does not act as a functional opposite to recreational screen time.

## Introduction

Physical activity (PA), described as “any bodily movement produced by skeletal muscles that results in energy expenditure^1^”, and low levels of sedentary time spent in front of screens and monitors (screen time, ST) have been associated with improved physical, psychosocial, and mental health^2–5^, especially among children and adolescents^6^. For a healthy lifestyle, the World Health Organization (WHO) and other authors recommend 60 min of moderate-to-vigorous daily PA for children and adolescents and low levels of recreational screen time (ST)^7,8^. Although there is no consensus yet for ST recommendations in children and adolescents, several experts have established guidelines recommending no more than 2 h of recreational ST for youth^9,10^.

Many countries have successfully worked towards these goals by implementing different organized, i.e., guided and repeated opportunities for being active, such as extracurricular sports classes in schools, and nonorganized opportunities such as public sports facilities or jogging routes in youth’s everyday life^9,11^. Therefore, the COVID-19 outbreak and concomitant shutdown of organized sports and public sports facilities in most countries resulted in crucial changes in youth’s daily routines and their opportunities for being active.

After the WHO had declared COVID-19 a global pandemic on March 11, 2020, the federal states in Germany closed kindergartens, schools, sports clubs, gyms, and other leisure institutions relevant to children’s and adolescents’ organized PA between March 16 and 18, 2020. The government also imposed physical distancing measures and contact restrictions, allowing no more than two people from different households to meet in public space^12^. This resulted in a complete shutdown of organized PA in the context of sports (SA). However, nonorganized SA, such as workouts at home, or jogging, and other forms of habitual PA besides sports (HA), like going for a walk or playing outside remained allowed if done alone or with people from the same household^13^. Although some stores were allowed to reopen on April 20, the lockdown was imposed until May 3, 2020^14^. An overview of the restrictions is presented in Fig. 2.

Previous studies indicate that healthy behaviors have complied more beneficially during structured days (especially school days) as compared to unstructured days (e.g., during vacations or on weekends)^15^. This is supported by data that shows the importance of structured institutions and organizations (e.g., schools, sports clubs) for children's and adolescents' SA in Germany^16^. Therefore, some researchers assume that the COVID-19 pandemic reinforces the pandemic of physical inactivity and sedentariness due to missing PA opportunities and physical distancing^17,18^ with a long return to normality^19^. Studies that have investigated the impact of COVID-19 on children’s PA and ST are scarce, but most of them support these assumptions. One study carried out in Shanghai compared data of 2426 children and adolescents (6–17 years) before and during the pandemic. The results showed that participants drastically reduced overall PA from an average of 540 to 105 min per week, while they increased ST from an average of 170 to 450 min weekly^20^. A study using prospective data from 823 adolescents around the age of 16 years in Croatia also showed that PA levels decreased and that there was a stronger decrease in adolescents living in urban areas^21^. Another study investigated step count changes in 109 children with congenital heart disease in Canada, showing that total steps decreased by 21–24% at the end of March and beginning of April 2020 as compared to the same time in 2019^22^. A study with 41 children between 6 and 18 years of age with obesity in Italy also showed that children reduced PA by almost 2.5 h per week^23^.

Two studies investigated the compliance with PA guidelines and ST recommendations. A study carried out in Shanghai showed that the compliance with the PA guidelines dropped from 60% before the pandemic to 17.7% during the pandemic^20^. Another study among 1472 children and adolescents between 5 and 17 years of age from Canada showed that 18% adhered to the PA guidelines and 11.2% adhered to the screen time recommendations^24^ during COVID-19. In comparison, Canada reported a 35% compliance with the PA guidelines and a 8–64% compliance with the ST guidelines in their PA report card of 2018^25^.

In sum, studies on the impact of COVID-19 on PA and ST lack methodological comparability, and the representativeness of most samples is low. Comparability between the different studies is also limited through the respective countries’ different policy measures that probably impacted PA and ST. Most countries reacted to the pandemic by a shutdown of real-world social life, including institutions that offered preventive health care with organized PA. However, the different reactions to the pandemic also created a patchwork of different natural experiments that may reveal interesting insights into human behavior. One important question derived from the circumstances in Germany is to what extend children and adolescents will transfer organized, guided PA to other forms of self-determined PA, or whether they will transfer it to ST and other sedentary behaviors instead.

In the following, we present the results of a natural experimental study among children and adolescents between 4 and 17 years of age in a nationwide representative sample during the strictest time of the first COVID-19 lockdown in Germany. We investigated how SA, HA, and recreational ST in children and adolescents in Germany changed during the COVID-19 lockdown using the data of two measurement points before (pre) and during (peri) the lockdown from the German Motorik-Modul (MoMo) cohort study.

## Results

**Figure 1:**
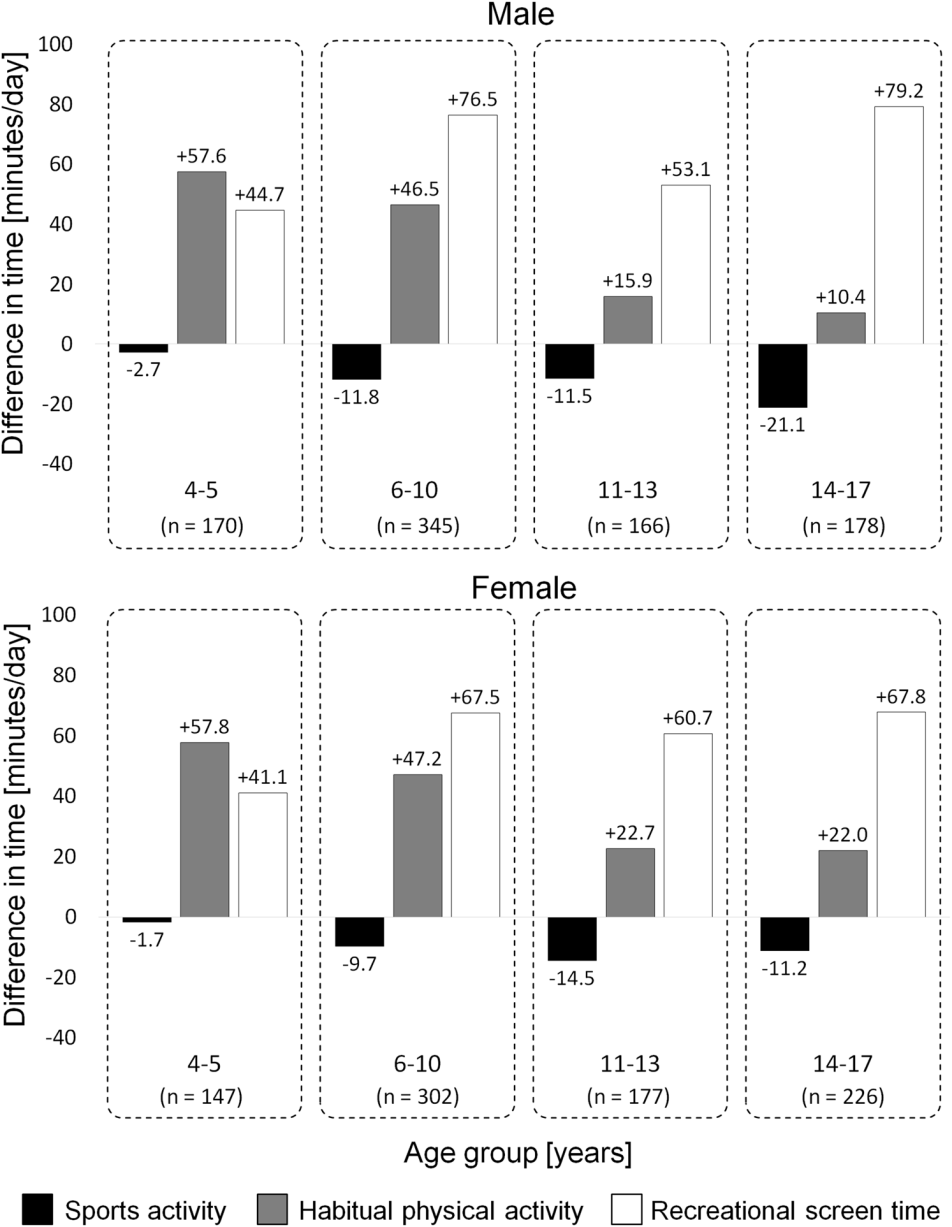
Differences for total amount of sports, habitual physical activity, and recreational screen time among youth in Germany pre and peri the COVID-19 lockdown (MoMo study).

The MoMo PA questionnaire measures PA and ST in different settings and allows to map PA recommendations^8^ and recommendations for recreational ST^7,8^. PA is further differentiated into different forms of organized and nonorganized SA and HA. An overview of differences between the pre and peri measurements of the whole sample of 4- to 17-year-olds can be found in Fig. 1.

### Guideline adherence

Table 1 shows how many days participants reported to be active for more than 60 min during a normal week pre and peri the lockdown and indicates the percentage of participants that adhered to the PA and ST guidelines. The analysis revealed a main effect of the lockdown on reported active days (F_1, 1686_ = 72.9, *p* < 0.01, p. η^2^ = 0.041) with an increase of 0.44 days per week. Additionally, an interaction between the lockdown and age group for active days was found (F_3, 1686_ = 4.5, *p* < 0.01, p. η^2^ = 0.008). 4- to 5-year-olds increased their PA by 0.76 active days whereas 14- to 17-year-olds increased it by 0.26 days. In terms of adherence to the WHO PA guidelines, this translates into a significant 11.1% overall increase during the lockdown with differences being larger for younger participants (4- to 5-year-olds + 14.7%) than for older ones (14- to 17-year-olds + 4.8%). At the same time, ST guideline adherence decreased by 17.5% (cf. Table 1).

**Table 1:** Overall physical activity and guideline adherence before and during the COVID-19 lockdown in Germany (MoMo Study).

Age	Sex	Days active [days/week]			PA guideline adherence			ST guideline adherence		
		Pre (M ± s)	Peri (M ± s)	Peri–pre (diff. [95% CI], d)	Pre (%)	Peri (%)	Peri–pre (diff. [95% CI])	Pre (%)	Peri (%)	Peri–pre (diff. [95% CI])
4–5 (n = 317)	m	4.7 ± 1.8	5.6 ± 1.7	+ 0.9 [0.6, 1.2], 0.46**	30.0	47.7	+ 17.7% [7.3, 27.5]**	94.7	69.4	− 25.3% [− 17.5, − 33.0]**
	f	5.0 ± 1.8	5.6 ± 1.6	+ 0.6 [0.3, 0.9], 0.30**	32.2	43.5	+ 11.4% [0.2, 22.1]*	92.5	76.9	− 15.6% [− 7.4, − 23.7]**
	Ø	4.8 ± 1.8	5.6 ± 1.6	+ 0.8 [0.5, 1.0], 0.39**	31.0	45.7	+ 14.7% [7.2, 22.1] **	93.7	72.9	− 20.1% [− 15.2, − 26.4]**
6–10 (n = 647)	m	4.8 ± 1.7	5.2 ± 1.9	+ 0.4 [0.2, 0.7], 0.19**	28.7	44.1	+ 15.4% [8.2, 22.4]**	77.6	42.4	− 35.5% [− 28.4, − 42.0]**
	f	4.5 ± 1.7	5.1 ± 1.8	+ 0.6 [0.4, 0.8], 0.29**	22.7	37.4	+ 14.8% [7.4, 21.8]**	79.7	52.6	− 27.1% [− 19.6, − 34.1]**
	Ø	4.7 ± 1.7	5.2 ± 1.9	+ 0.5 [0.3, 0.7], 0.24**	25.9	41.0	+ 15.1% [10.0, 20.1]**	78.6	47.1	− 31.6% [− 26.5, − 36.4]**
11–13 (n = 343)	m	4.0 ± 1.8	4.3 ± 2.0	+ 0.3 [0.0, 0.6], 0.15	15.2	23.5	+ 8.3% [− 0.2, 16.8]	38.7	15.7	− 23.0% [− 13.5, − 32.0]**
	f	3.6 ± 1.6	3.8 ± 2.0	+ 0.3 [− 0.1, 0.6], 0.13	6.9	14.1	+ 7.2% [0.8, 13.8]*	41.4	17.0	− 24.3% [− 14.9, − 33.2]**
	Ø	3.8 ± 1.7	4.1 ± 2.0	+ 0.3 [0.1, 0.5], 0.14*	10.9	18.7	+ 7.7% [2.4, 13.1]*	40.1	16.4	− 23.7% [− 17.0, − 30.1]**
14–17 (n = 404)	m	3.7 ± 1.6	3.7 ± 1.8	+ 0.1 [− 0.3, 0.4], 0.02	5.7	11.2	+ 5.6% [− 0.3, 11.6]	15.3	7.3	− 8.3% [− 1.4, − 14.8]*
	f	3.5 ± 1.6	3.9 ± 1.8	+ 0.4 [0.2, 0.7], 0.22**	5.9	9.7	+ 3.9% [− 1.2, 9.0]	30.8	16.8	− 14.0% [− 6.1, − 21.7]**
	Ø	3.6 ± 1.6	3.8 ± 1.8	+ 0.3 [0.1, 0.5], 0.13*	5.8	10.4	+ 4.8% [0.9, 8.5]*	23.9	12.6	− 11.3% [− 6.0, − 16.6]**
4–17 (n = 1711)	m	4.4 ± 1.8	4.8 ± 2.0	+ 0.4 [0.3, 0.6], 0.20**	21.4	34.0	+ 12.5% [8.2, 16.6]**	60.7	35.2	− 25.5% [− 20.8, − 30.0]**
	f	4.1 ± 1.8	4.6 ± 1.9	+ 0.5 [0.3, 0.6], 0.24**	16.4	26.3	+ 9.5% [4.5, 13.3]**	61.1	40.0	− 21.1% [− 16.4, − 25.7]**
	Ø	4.3 ± 1.8	4.7 ± 2.0	+ 0.4 [0.4, 0.5], 0.22**	19.1	30.2	+ 11.1% [8.2, 13.9]**	60.9	37.6	− 23.3% [− 20.0, − 26.5]**

PA guideline adherence: being physically active for 60 min or more a day during a normal week; ST guideline adherence: Using recreational screen time media for 120 min or less a day during a normal week.

*m* male, *f* female, *Ø* mean of males and females, *M* mean, *s* standard deviation, *diff.* difference peri-pre, *95% CI* 95% confidence interval, *d* effect size Cohen’s d.

*^/^**Significant difference (*p < .05; **p < .01).

### Sports activity

Organized sports at school and in sports clubs were shut down during the lockdown which leads to no measurable organized sports during the peri measurement. Table 2 shows the average daily minutes spent doing organized and nonorganized sports, and the total amount of sports pre and peri the lockdown. A significant main effect of the lockdown on nonorganized sports was found (F_1, 1601_ = 626.3, *p* < 0.01, p. η^2^ = 0.281). Participants reported an increase in nonorganized sports of 17.7 min per day. Additionally, an interaction between the lockdown and age was found for nonorganized sports (F_3, 1601_ = 9.8, *p* < 0.01, p. η^2^ = 0.018). 14- to 17-year-olds increased their nonorganized sports by 17.0 min per day whereas 4- to 5-year-olds increased it by 11.8 min. For the total amount of sports, we found a main effect of the lockdown (F_1, 1644_ = 122.3, *p* < 0.01, p. η^2^ = 0.069) in a negative direction. Participants reported a decrease in the total amount of sports of 10.8 min per day, and an interaction between the lockdown and age (F_3, 1644_ = 8.6, *p* < 0.01, p. η^2^ = 0.015) was significant: 14- to 17-year-olds reduced their total amount of sports by 15.6 min per day whereas 4- to 5- year-olds reduced it by 2.2 min.

**Table 2:** Sports activity before and during the COVID-19 lockdown in Germany (MoMo Study).

Age	Sex	Organized sports^a^ [minutes per day]		Nonorganized sports [minutes per day]		Total amount of sports [minutes per day]		
		Pre (M ± s)	Peri (M ± s)	Pre (M ± s)	Peri (M ± s)	Pre (M ± s)	Peri (M ± s)	Peri–pre (diff. [95% CI], d)
4–5 (n = 317)	m	13.7 ± 9.4	0.0 ± 0.0	3.3 ± 7.5	14.3 ± 29.4	16.8 ± 11.3	14.3 ± 29.4	− 2.7 [− 2.4, 2.0], 0.09
	f	14.0 ± 11.8	0.0 ± 0.0	3.5 ± 6.2	16.2 ± 29.3	17.5 ± 14.0	16.2 ± 29.3	− 1.7 [− 6.9, 3.6], 0.05
	Ø	13.8 ± 10.6	0.0 ± 0.0	3.4 ± 6.9	15.2 ± 29.3	17.1 ± 12.6	15.2 ± 29.3	− 2.2 [− 5.6, 1.2], 0.07
6–10 (n = 647)	m	31.9 ± 19.5	0.0 ± 0.0	4.8 ± 13.1	24.6 ± 38.5	36.5 ± 25.5	24.6 ± 38.5	− 11.8 [− 16.4, − 7.2], 0.28**
	f	25.5 ± 17.3	0.0 ± 0.0	4.7 ± 8.1	21.0 ± 33.0	30.2 ± 18.2	21.0 ± 33.0	− 9.7 [− 13.5, − 5.9], 0.30**
	Ø	28.9 ± 18.8	0.0 ± 0.0	4.7 ± 11.1	23.0 ± 36.1	33.6 ± 22.6	23.0 ± 36.1	− 10.8 [− 13.8, − 7.8], 0.28**
11–13 (n = 343)	m	37.6 ± 22.2	0.0 ± 0.0	6.4 ± 12.6	32.8 ± 44.7	44.3 ± 25.7	32.8 ± 44.7	− 11.5 [− 17.7, − 5.2], 0.29**
	f	34.1 ± 25.5	0.0 ± 0.0	7.1 ± 15.6	26.7 ± 36.9	41.1 ± 28.1	26.7 ± 36.9	− 14.5 [− 20.7, − 8.3], 0.36**
	Ø	35.8 ± 24.0	0.0 ± 0.0	6.7 ± 14.2	29.7 ± 40.9	42.6 ± 27.0	29.7 ± 40.9	− 13.0 [− 17.4, − 8.6], − 0.32**
14–17 (n = 404)	m	36.1 ± 27.5	0.0 ± 0.0	12.4 ± 19.2	26.7 ± 39.6	47.4 ± 30.5	26.7 ± 39.6	− 21.1 [− 27.2, − 15.1], 0.53**
	f	30.7 ± 25.2	0.0 ± 0.0	11.7 ± 19.4	30.9 ± 32.0	42.3 ± 29.8	30.9 ± 32.0	− 11.2 [− 15.7, − 6.8], 0.34**
	Ø	33.1 ± 26.3	0.0 ± 0.0	12.0 ± 19.3	29.0 ± 25.6	44.6 ± 30.2	29.0 ± 25.6	− 15.6 [− 19.3, − 12.0], 0.43**
4–17 (n = 1711)	m	30.3 ± 22.2	0.0 ± 0.0	6.3 ± 14.0	24.6 ± 38.8	36.3 ± 26.8	24.6 ± 38.8	− 11.8 [− 14.5, − 9.1], 0.30**
	f	26.7 ± 21.8	0.0 ± 0.0	6.9 ± 13.8	24.0 ± 33.4	33.5 ± 25.1	24.0 ± 33.4	− 9.7 [− 12.1, − 7.3], 0.28**
	Ø	28.5 ± 22.1	0.0 ± 0.0	6.6 ± 13.9	24.3 ± 36.2	34.9 ± 26.0	24.3 ± 36.2	− 10.8 [− 12.6, − 9.0], 0.29**

*m* male, *f* female, *Ø* mean of males and females, *M* mean, *s* standard deviation, *95% CI* 95% confidence interval; d: effect size Cohen’s d.

*^/^**Significant difference (*p < .05; **p < .01).

^a^Organized sports were completely shut-down during the study.

### Habitual physical activity

In the pre-measurement, significant effects of age were detected for playing outside (F_3, 1665_ = 166.7, *p* < 0.01, p. η^2^ = 0.231), walking and cycling (F_3, 1554_ = 12.1, *p* < 0.01, p. η^2^ = 0.023), gardening (F_3, 1652_ = 7.1, *p* < 0.01, p. η^2^ = 0.013), housework (F_3, 1658_ = 38.3, *p* < 0.01, p. η^2^ = 0.065), and the total amount of HA (F_3, 1680_ = 42.3, *p* < 0.01, p. η^2^ = 0.070) (cf. Table 3). Younger children accumulated more minutes of PA playing outside whereas adolescents accumulated more minutes walking and cycling as well as gardening and doing housework. In sum, with 136.5 min per day, 4- to 5-year-olds accumulated more minutes of daily HA than 14- to 17-year-olds with 80.3 min. Furthermore, significant effects of sex were detected for playing outside (F_1, 1665_ = 12.6, *p* < 0.01, p. η^2^ = 0.007), gardening (F_1, 1652_ = 14.1, *p* < 0.01, p. η^2^ = 0.008), housework (F_1, 1658_ = 10.4, *p* < 0.01, p. η^2^ = 0.006), and the total amount of HA (F_1, 1680_ = 11.9, *p* < 0.01, p. η^2^ = 0.007) in the pre-measurement. Boys accumulated more minutes of PA by playing outside, walking and cycling, and gardening, whereas girls accumulated more minutes of PA doing housework.

**Table 3:** Habitual physical activity before and during the COVID-19 lockdown in Germany (MoMo Study).

Age	Sex	Playing outside [minutes per day]		Walking and cycling [minutes per day]		Gardening [minutes per day]		Housework [minutes per day]		Total amount of habitual activity [minutes per day]		
		Pre (M ± s)	Peri (M ± s)	Pre (M ± s)	Peri (M ± s)	Pre (M ± s)	Peri (M ± s)	Pre (M ± s)	Peri (M ± s)	Pre (M ± s)	Peri (M ± s)	Peri–pre (diff. [95% CI], d)
4–5 (n = 317)	m	92.4 ± 57.3	131.8 ± 104.9	36.7 ± 27.6	43.4 ± 30.2	8.4 ± 16.0	12.1 ± 21.2	3.6 ± 6.0	5.9 ± 13.1	136.5 ± 70.1	194.1 ± 128.3	+ 57.6 [37.8, 77.4], 0.44**
	f	83.3 ± 58.1	129.5 ± 96.6	37.7 ± 25.8	40.8 ± 26.0	6.3 ± 12.7	10.1 ± 23.0	5.1 ± 6.7	6.5 ± 9.4	129.5 ± 76.3	187.3 ± 113.2	+ 57.8 [36.7, 79.0], 0.45**
	Ø	88.2 ± 57.8	130.8 ± 100.9	37.2 ± 26.7	42.2 ± 28.3	7.4 ± 14.6	11.2 ± 22.0	4.3 ± 6.4	6.2 ± 11.5	133.3 ± 73.0	191.0 ± 121.5	+ 57.7 [43.3, 72.1], 0.45**
6–10 (n = 647)	m	77.2 ± 58.3	108.5 ± 89.2	38.3 ± 24.1	40.9 ± 30.9	5.6 ± 12.5	11.2 ± 26.2	4.3 ± 5.7	8.3 ± 14.4	122.7 ± 72.7	169.1 ± 111.3	+ 46.5 [34.9, 58.0], 0.43**
	f	72.6 ± 58.4	102.6 ± 89.3	34.3 ± 23.0	40.6 ± 27.8	5.1 ± 15.3	8.9 ± 19.8	5.8 ± 8.0	11.4 ± 20.8	116.2 ± 77.3	163.4 ± 109.5	+ 47.2 [35.4, 58.9], 0.46**
	Ø	75.0 ± 58.3	105.7 ± 89.2	36.4 ± 23.6	40.8 ± 29.5	5.3 ± 13.9	10.1 ± 23.4	5.0 ± 7.0	9.7 ± 17.8	119.6 ± 74.9	166.4 ± 110.4	+ 46.8 [38.6, 55.0] 0.44**
11–13 (n = 343)	m	50.6 ± 54.7	55.0 ± 68.5	39.4 ± 27.4	38.4 ± 27.4	6.5 ± 20.3	13.5 ± 21.3	7.6 ± 12.1	11.4 ± 19.5	102.8 ± 77.7	118.7 ± 98.1	+ 15.9 [1.3, 30.5], 0.17*
	f	33.2 ± 39.0	39.9 ± 53.0	36.6 ± 21.1	39.4 ± 30.1	4.2 ± 12.7	9.3 ± 23.0	9.1 ± 10.3	16.4 ± 19.7	81.2 ± 55.2	104.0 ± 81.4	+ 22.7 [11.5, 34.0], 0.31**
	Ø	41.8 ± 48.1	47.3 ± 61.5	38.0 ± 24.4	38.9 ± 29.3	5.3 ± 16.9	11.4 ± 27.4	8.4 ± 11.2	14.0 ± 19.7	91.8 ± 68.5	111.1 ± 90.2	+ 19.4 [10.3, 28.5], 0.23**
14–17 (n = 404)	m	16.2 ± 34.6	18.7 ± 40.2	47.7 ± 33.8	39.5 ± 34.6	16.0 ± 43.5	28.6 ± 63.5	11.4 ± 16.5	13.5 ± 17.5	89.1 ± 74.4	99.5 ± 99.4	+ 10.4 [-3.7, 24.5], 0.11
	f	10.2 ± 22.9	11.4 ± 22.8	44.3 ± 24.2	44.4 ± 33.0	5.6 ± 11.3	18.4 ± 48.8	16.4 ± 30.2	20.3 ± 22.9	73.4 ± 56.9	95.4 ± 82.6	+ 22.0 [11.8, 32.2], 0.28**
	Ø	12.9 ± 28.8	14.6 ± 31.9	45.8 ± 28.9	42.2 ± 33.8	10.2 ± 30.5	22.9 ± 55.9	14.2 ± 25.1	17.3 ± 20.9	80.3 ± 65.6	97.2 ± 90.3	+ 16.9 [8.5, 25.3], 0.20**
4–17 (n = 1711)	m	63.0 ± 59.7	85.0 ± 91.5	40.2 ± 28.0	40.5 ± 31.1	8.5 ± 24.6	15.5 ± 38.0	6.3 ± 10.7	9.5 ± 16.2	114.6 ± 75.5	149.8 ± 115.5	+ 35.2 [27.9, 42.6], 0.32**
	f	50.7 ± 55.9	71.5 ± 85.5	38.1 ± 23.7	41.4 ± 29.5	5.2 ± 13.4	11.7 ± 31.5	9.2 ± 17.8	14.0 ± 20.3	99.8 ± 71.4	137.0 ± 104.8	+ 37.2 [30.6, 42.8], 0.38**
	Ø	56.9 ± 58.2	78.3 ± 88.8	39.2 ± 26.0	41.0 ± 30.3	6.9 ± 19.9	13.6 ± 35.0	7.8 ± 14.7	11.8 ± 18.5	107.3 ± 73.9	143.5 ± 110.5	+ 36.2 [31.3, 41.2], 0.35**

*m* male, *f* female, *Ø* mean of males and females, *M* mean, *s* standard deviation, *95% CI* 95% confidence intervals, *d* effect size Cohen’s d.

*^/^**Significant difference (*p < .05; **p < .01).

The longitudinal analysis revealed a main effect of the lockdown on playing outside (F_1, 1665_ = 12.6, *p* < 0.01, p. η^2^ = 0.007), gardening (F_1, 1638_ = 80.7, *p* < 0.01, p. η^2^ = 0.047), housework (F_1, 1645_ = 62.2, *p* < 0.01, p. η^2^ = 0.036), and the total amount of HA (F_1, 1673_ = 181.4, *p* < 0.01, p. η^2^ = 0.098). No main effect of the lockdown was found for walking and cycling. Playing outside increased by 21.4 min, gardening increased by 6.7 min, housework increased by 4.0 min, and the total amount of HA increased by 36.2 min. An interaction between the lockdown and age was found for playing outside (F_3, 1654_ = 22.2, *p* < 0.01, p. η^2^ = 0.039), gardening (F_3, 1638_ = 7.3, *p* < 0.01, p. η^2^ = 0.013), housework (F_3, 1645_ = 2.8, *p* = 0.04, p. η^2^ = 0.003), and the total amount of HA (F_3, 1673_ = 14.9, *p* < 0.01, p. η^2^ = 0.026) (c.f. Table 3).

### Recreational screen time

Significant effects of age were detected for watching TV (F_3, 1671_ = 7.7, *p* < 0.01, p. η^2^ = 0.014), gaming (F_3, 1660_ = 147.5, *p* < 0.01, p. η^2^ = 0.210), recreational internet usage (F_3, 1655_ = 410.0, *p* < 0.01, p. η^2^ = 0.426), and the total amount of recreational ST (F_3, 1652_ = 257.9, *p* < 0.01, p. η^2^ = 0.316) during the pre-study (c.f. Table 4). Significant effects for sex were found for gaming (F_1, 1660_ = 123.4, *p* < 0.01, p. η^2^ = 0.069) and the total amount of recreational ST (F_1, 1652_ = 21.8, *p* < 0.01, p. η^2^ = 0.013). Significant interactions between age and sex for gaming (F_3, 1660_ = 52.4, *p* < 0.01, p. η^2^ = 0.086) and the total amount of recreational ST (F_3, 1652_ = 12.3, *p* < 0.01, p. η^2^ = 0.022) indicate that differences between the sexes increase with age (c.f. Table 4).

**Table 4:** Recreational screen time usage before and during the COVID-19 lockdown in Germany (MoMo Study).

Age	sex	TV [minutes per day]		Gaming [minutes per day]		Recreational Internet [minutes per day]		Total amount of recreational screen time [minutes per day]		
		Pre (M ± s)	Peri (M ± s)	Pre (M ± s)	Peri (M ± s)	Pre (M ± s)	Peri (M ± s)	Pre (M ± s)	Peri (M ± s)	Peri–pre (diff. [95% CI], d)
4–5 (n = 317)	m	38.8 ± 29.8	62.9 ± 39.4	7.2 ± 16.6	19.9 ± 28.2	7.1 ± 16.4	14.5 ± 30.7	52.7 ± 47.8	97.4 ± 64.8	+ 44.7 [36.5, 52.8], 0.83**
	f	36.5 ± 30.2	57.7 ± 42.4	7.0 ± 18.9	15.9 ± 28.3	7.4 ± 13.1	15.3 ± 28.0	51.4 ± 47.7	88.4 ± 64.2	+ 37.0 [29.4, 44.6], 0.80**
	Ø	37.7 ± 29.9	60.5 ± 40.9	7.1 ± 17.6	18.0 ± 28.3	7.2 ± 14.9	14.8 ± 29.4	52.1 ± 47.7	93.2 ± 64.6	+ 41.1 [35.5, 46.7], 0.81**
6–10 (n = 647)	m	47.1 ± 37.2	67.4 ± 50.7	26.6 ± 33.5	58.8 ± 54.2	15.4 ± 28.3	39.4 ± 51.6	88.7 ± 72.4	165.2 ± 111.7	+ 76.5 [66.5, 86.4], 0.82**
	f	47.1 ± 35.8	68.3 ± 48.7	19.8 ± 31.8	36.2 ± 47.5	19.9 ± 39.1	38.7 ± 47.6	86.1 ± 82.1	143.2 ± 106.7	+ 57.2 [48.5, 65.8], 0.76**
	Ø	47.1 ± 36.5	67.8 ± 49.7	23.5 ± 32.9	48.3 ± 52.4	17.5 ± 33.8	39.1 ± 49.8	87.5 ± 77.0	154.9 ± 109.9	+ 67.5 [60.8, 74.2], 0.78**
11–13 (n = 343)	m	50.8 ± 53.3	57.5 ± 58.0	72.6 ± 63.0	101.7 ± 70.8	76.5 ± 70.4	93.4 ± 67.5	199.1 ± 145.2	252.1 ± 136.2	+ 53.1 [33.2, 73.0], 0.41**
	f	57.5 ± 58.0	81.0 ± 65.0	49.9 ± 58.3	65.1 ± 72.1	77.7 ± 64.3	106.3 ± 68.8	183.2 ± 127.1	251.2 ± 137.2	+ 67.9 [49.9, 86.0], 0.57**
	Ø	54.1 ± 53.4	69.7 ± 62.7	61.0 ± 61.6	82.9 ± 73.7	77.1 ± 67.2	100.0 ± 68.4	251.6 ± 136.5	251.6 ± 136.5	+ 60.7 [47.4, 74.1], 0.49**
14–17 (n = 404)	m	45.7 ± 55.4	68.3 ± 74.0	102.6 ± 71.4	137.0 ± 78.5	116.5 ± 71.1	138.9 ± 70.4	264.6 ± 138.0	343.8 ± 152.5	+ 79.2 [55.9, 102.4], 0.51**
	f	44.5 ± 49.0	71.7 ± 64.7	29.7 ± 50.8	45.6 ± 73.6	115.3 ± 68.9	130.4 ± 72.1	188.8 ± 121.1	247.5 ± 142.2	+ 58.7 [41.7, 75.7], 0.46**
	Ø	45.0 ± 51.8	70.2 ± 68.9	61.9 ± 70.7	86.0 ± 88.3	115.8 ± 70.0	134.2 ± 71.4	222.4 ± 134.1	290.2 ± 154.3	+ 67.8 [53.8, 81.7], 0.48**
4–17 (n = 1711)	m	45.9 ± 34.8	64.8 ± 55.9	47.4 ± 59.8	75.6 ± 72.2	47.1 ± 65.6	66.2 ± 72.4	139.2 ± 130.4	205.4 ± 146.8	+ 66.2 [58.6, 73.7], 0.59**
	f	46.6 ± 43.3	70.0 ± 56.4	26.4 ± 44.7	41.2 ± 60.7	55.1 ± 67.7	73.1 ± 73.4	127.3 ± 115.0	183.6 ± 134.6	+ 56.3 [9.6, 63.0], 0.57**
	Ø	46.2 ± 43.5	67.4 ± 56.2	37.0 ± 54.9	58.5 ± 68.9	51.1 ± 66.8	69.6 ± 73.0	133.3 ± 123.1	194.5 ± 141.3	+ 61.2 [56.2, 66.3], 0.58**

*m* male, *f* female, *Ø* mean of males and females, *M* mean, *s* standard deviation, *95% CI* 95% confidence intervals for complex samples, *d* effect size Cohen’s d.

*^/^**Significant difference (*p < .05; **p < .01).

A main effect of the lockdown is significant for watching television (F_1, 1667_ = 248.8, *p* < 0.01, p. η^2^ = 0.130), gaming (F_1, 1654_ = 209.5, *p* < 0.01, p. η^2^ = 0.112), recreational internet usage (F_1, 1646_ = 149.9, *p* < 0.01, p. η^2^ = 0.083), and the total amount of recreational ST (F_1, 1676_ = 494.7, *p* < 0.01, p. η^2^ = 0.228). TV watching increased by 21.2 min, gaming increased by 21.5 min, recreational internet usage increased by 18.5 min, and the total amount of recreational ST increased by 61.2 min per day. An interaction between the lockdown and the participants’ age was found for internet usage (F_3, 1646_ = 4.9, *p* < 0.01, p. η^2^ = 0.009), gaming (F_3, 1654_ = 4.9, *p* < 0.01, p. η^2^ = 0.009), and the total amount of recreational ST (F_3, 1676_ = 5.2, *p* < 0.01, p. η^2^ = 0.009). Interaction effects between the lockdown and sex were only found for gaming (F_3, 1654_ = 20.9, *p* < 0.01, p. η²= 0.012). This translates into a 17.5% overall decrease in adherence to the recreational ST guideline during the lockdown with a substantially larger decrease for participants aged 14–17 years with − 18.4% compared to 4- to 5-year-olds with − 4.1%.

## Discussion

Due to consecutive tracking of the PA, fitness, and health of children and adolescents in Germany since 2003, the MoMo study had the opportunity to rely on longitudinal PA data from a representative national cohort directly before and during the COVID-19 pandemic.

Our results show a decline in SA among boys and girls of all age groups paralleled by an increase in HA and recreational ST. In summary, the negative effects of the lockdown were stronger pronounced among adolescence, which showed a larger decline of SA and a lower increase in HA compared to younger children. A decline in SA is in line with other studies that tracked PA during the COVID-19 lockdown by questionnaires in Canada^24,26,^ China^20^, Spain^27^, as well as by step counts in Italy^23^ and Canada^22^. In contrast to SA, the adherence to the PA guidelines and total PA increased among children and adolescents in Germany, which is in line with a study from Belgium^28^ but in contrast to studies from other countries^20–24,26,27^. The different behaviors of children and adolescents among countries may be related to different context factors such as different policy restrictions and the number of COVID-19 infections among countries that directly affected behavior. For example, during the lockdown in China, outdoor exercises were not allowed^29^, whereas Belgium had only mild restrictions. On the other hand, we assume that differences may be caused by methodological issues of tracking PA. For example, the questionnaire we used to track PA differentiated between a multitude of settings and activities and therefore we were able to analyze and contrast shifts among different types of PA and different settings.

In our sample, the increase in guideline adherence, which stands in contrast to the 28.5 min decline in organized SA, was not only initiated by an increase in nonorganized SA of 17.7 min per day but ultimately achieved by a 36.2 min increase in daily HA. Data from the US confirm that nonorganized PA and outdoor play were highly relevant during the COVID-19 pandemic, especially among younger children^30^. We found a change in total daily PA from 142.2 min pre to 167.8 min peri the lockdown in our study. In contrast, the study among 6- to 17-year-olds from Shanghai reported a change in total daily PA from 77.1 min pre to 15.0 min peri the lockdown^20^. PA behavior is a multi-dimensional construct with fluid settings. The different reactions to the pandemic all over the world showed that many children, adolescents, and adults can compensate for the elimination of settings within weeks. Nevertheless, being able to go outdoors safely with rules of social distancing and contact to at least one known person was the most striking difference in the execution of lockdowns between countries and seems crucial in maintaining sufficient levels of PA among children and adolescents.

Interestingly, an increase in nonorganized sports was not limited to those who were already doing sports before the lockdown in our study. 30.2% of those who had reported no SA before the lockdown engaged in nonorganized sports during the lockdown whereas only 60.3% of those who had engaged in SA in sports clubs before the lockdown transferred their organized to nonorganized SA. Within-person analyses pre to peri the lockdown showed that the mean correlation coefficients were substantially lower for nonorganized sports (r = 0.13, *p* < 0.01) compared to HA (r = 0.43; *p* < 0.01), days active (r = 0.41; *p* < 0.01), or recreational ST (r = 0.69; *p* < 0.01). These results are supported by a cross-sectional study with 13,515 adults from Belgium^28^. The data from Belgium shows a general increase in exercise frequencies, but exercise response to the lockdown differed between active and nonactive people before the lockdown: Of those who had exercised at least once a week before the lockdown, 23% reported less and 36% reported more exercising during the lockdown. In contrast, 58% of those who had exercised less than once a week before the lockdown reported more exercising during the lockdown^28^. Most likely, a more pronounced focus on a healthy lifestyle during the pandemic triggered this behavior in countries where policy restrictions allowed exercising. We also analyzed the distribution of PA and ST among a multitude of socioeconomic variables and found a slightly smaller impact of the socioeconomic status on PA and ST peri compared to pre lockdown. This may be explained by the fact that children and adolescents from families with lower socioeconomic status engage less often in organized and more often in nonorganized SA compared to those from households with a higher socioeconomic status^11^. The Gini coefficient^31^, which has recently been used to describe inequalities among the PA distribution between countries^32^, decreased from 0.80 to 0.70 for SA and from 0.47 to 0.39 for recreational ST but increased from 0.35 to 0.40 for HA among children and adolescents during the lockdown in Germany. A decrease in the Gini coefficient for SA and ST can be interpreted as a step towards a more equally distributed amount of SA and ST among children and adolescents as entry barriers to organized SA played no role and the demand for a healthy lifestyle was high during the lockdown. We can derive from this that countries and policies need to enable access to nonorganized PA to all children and adolescents during potential future lockdowns. Especially for adolescents, internet interventions from sports clubs are one potential option but scientists should also focus on upcoming affordable virtual and augmented-reality solutions to enable comprehensive PA opportunities for children and adolescents from all social classes, especially in urban areas. In reaction to the drastic decrease in PA among children and adolescents in China^20^, authors from the Shanghai University of Sport recommended exercising at home but also mentioned the danger of prolonged stays at home which can increase behaviors that lead to inactivity and contribute to anxiety and depression^29^. In the context of the studies from other countries mentioned before, results speak for the fact that the current technical possibilities are not adequate to comprehensively maintain sufficient levels of PA at home. Until there are virtual options for PA that satisfy the needs of users to an extent that they are comparably motivational and continual to analog forms of PA, policies should focus on providing safe natural space for nonorganized outdoor activities in addition to organized offers.

The finding that children and adolescents in Germany spent more active time peri than pre lockdown may be explained by the simple fact that they had more recreational time to do so, but also theoretically by self-determination theory^33^ and a more pronounced focus on health. Studies show that boys get most of their moderate-to-vigorous PA at school during recess and non-structured times^34^. During non-structured days, children and adolescents can choose PA behaviors that allow them to develop mastery, skills, and competence and can satisfy the need that they are in control of their behavior which leads to more motivation for PA^34^.

The increase in recreational ST among children and adolescents in Germany is in line with data about leisure screen time in Spain^27^ and China^20^. The study from Spain found an increase in ST between 132 (3–5-year-olds) and 198 (13–16-year-olds) minutes per day and a significant increase in overall sleep time among the 13–16-year-olds of 36 minutes per day during strict COVID-19 confinement. The Chinese study shows an increase in daily recreational ST among 6- to 17-year-olds from 24.3 to 64.3 min and an increase in daily total ST from 87.1 to 334.3 min. Adding nonrecreational screen media usage to our data, we find an increase from 146.1 to 237.5 min in total ST pre to peri the lockdown and an increase from 215.1 to 338.7 min in daily total media usage (including reading and listening to music). Home confinement restricted opportunities for children in China more than in Germany, and the fact that, as of reports by free journalists^35^, homeschooling started much earlier in China (March 2, 2020) than in Germany (May, 4, 2020) explains the pronounced increase in total ST in China. The numerically larger increase in ST with increasing age we found is supported by a German study that interviewed more than 8000 parents of children aged 3–15 years during the lockdown and found an increase of 38% (kindergarten) to 72% (secondary education) among recreational ST^36^. An increase in ST paralleled by an increase in PA among children and adolescents in Germany during the COVID-19 lockdown adds a piece to the puzzle of the relationship between PA and ST. Although it may be against common understanding at first glance that PA and ST are mutually exclusive in youth’s recreational time, a meta-analysis of 163 studies about the associations between sedentary behavior and physical activity in children and adolescents found a global correlation of only r = 0.11 between PA and ST^37^. The authors stated that the activities do not behave as functional opposites and substitute each other only marginally. Cluster analyses of the MoMo baseline sample confirmed that there are a multitude of different behavioral patterns concerning PA and recreational ST including children and adolescents with very large amounts of PA and ST and some that do not report any PA but also report low amounts of ST at the same time^38,39^. Other studies support the idea of ST and physical inactivity being different risk factors for an unhealthy lifestyle^40–42^. In the cluster-randomized, controlled trial “Switch-Off 4 Healthy Minds”, the authors found that a successful reduction of ST did not lead to a meaningful increase in PA among adolescents^43^.

Despite the comprehensive approach of tracking PA in Germany within the MoMo study, there are some limitations to this study. First, all our results are based on self-reports. Our questionnaire has been tailored to the different PA settings in Germany with a higher focus on recall bias compared to the relatively short and unspecific, yet economical, GPAQ^44^, which has been used for example in the Chinese study^20^. We also use device-based measures (Actigraph GT3X) to validate and augment our PA data in MoMo, but decided that it is not ethically justifiable to use them on a large sample during the lockdown. Device-based studies of PA during the lockdown are scarce and the little data that have been published so far rely on nonrepresentative ad-hoc samples, for example, children with congenital heart disease^22^ or already active people^45^. The largest sources of published device-based measured PA peri the lockdown are country-stratified data from Garmin wearables^45^ and a smartphone app^46^. The Garmin data show that different activities such as walking or outdoor and indoor cycling changed differently among countries, with most of them increasing^45^. The study which analyzed step counts from a smartphone app confirmed substantial differences between countries but reported decreases in step counts within 30 days of the pandemic from 6.9% in Sweden to 48.7% in Italy with a mean decrease of 27.3%^46^. Second, as it is a natural experiment, there is no control group and we can only assume that the lockdown was causal for the changes in PA and ST behavior. Since a full representative MoMo study takes 2 years to be completed, the mean age peri exceeded the mean age pre by almost exactly 1 year (Table 5). Since studies show that especially nonorganized PA declines during maturation in humans^9,16,47,48^ as well as in most nonhuman species^49^, this may have led to a small underestimation of the observed increase in PA in our study.

**Table 5:** Sample characteristics.

	4- to 5-year-olds	6- to 10-year-olds	11- to 13-year-olds	14- to 17-year-olds	4- to 17-year-olds
Total [n]	317	647	343	404	1711
Boys [n (%)]	170 (53.6%)	345 (53.3%)	166 (48.4%)	178 (44.1%)	859 (50.2%)
Girls [n (%)]	147 (46.4%)	302 (46.7%)	177 (51.6%)	226 (55.9%)	852 (49.8%)

	M ± s	M ± s	M ± s	M ± s	M ± s
Age pre (years)	5.04 ± 0.55	8.35 ± 1.43	12.51 ± 0.89	15.93 ± 1.15	10.36 ± 4.04
Age peri (years)	6.03 ± 0.72	9.33 ± 1.51	13.52 ± 0.97	16.88 ± 1.27	11.34 ± 4.06

*M* mean; *s* standard deviation.

The weather was untypically warm in April 2020 in Germany. A mean temperature of 10.4 °C (9.6 °C in 2019) and on average 292.4 sunshine hours (227.9 in 2019) were measured across Germany in April, 2020^50,51^. Warmer temperatures are associated with more PA and less ST in mild climates^52^. We also analyzed our data for seasonal effects. When considering only those MoMo participants who were tested in April 2019, the effects on PA and recreational ST were stable. Nonorganized sports increased by 15.9 min per day, HA by 46.2 min per day, recreational ST by 49.5 min per day, and WHO guideline compliance increased from 17.6 to 33.7%.

Lastly, the representativeness of our longitudinal pre-peri sample is limited because of the unforeseen COVID-19 outbreak during the collection of the representative pre-study sample. However, 120 out of 167 initial sample points across Germany were reached during the pre-study. Since the survey design includes a circle concept which ensures that sample points with different environmental contexts are tested during different months of the year, the reduction in representativeness because of missing sample points can be considered as small. The main effects of the lockdown remained stable after stratifying the sample by potential influencing socioeconomic factors such as housing situation, region within Germany, socioeconomic status, and the number of siblings.

What we can conclude from this natural experiment is that in some individuals the insufficient levels of PA are context-driven (e.g. too much forced sitting during school, homework, and other organized sedentary activities) and will vanish, once they regain autonomy over their everyday life. It is striking that in countries with suitable circumstances like in Belgium^28^, people found their way to PA during the lockdown. Phases with high alertness and focus on health may be a chance to positively interact with people's preventive behavior.

The fact that some children and adolescents did not engage in PA peri the lockdown and that the decline in SA is more pronounced in older age groups is alarming. Among European countries, there is a weak agreement that total PA among children and adolescents declined during the 1980/1990s, however comparable data with high standards regarding the methodology is missing among studies from that time^47^. This trend is followed by a slight increase in PA and a substantial increase in ST during the 2000s in Germany^16,53,54^ and most European countries^48,55^, leading to the stagnation of total PA with a shift from nonorganized to organized settings during the 2010s^11^. The fact that the recommended 60 min of daily moderate to vigorous PA nearly doubled among children and adolescents during the COVID-19 lockdown after having been stable for two decades is striking and needs to be analyzed further from a scientific yet political point of view.

The special circumstances during the COVID-19 lockdown also told us that PA and recreational ST behavior do not act as functional opposites and may decrease or increase concurrently^43^. Although there are cases where youth show very high levels of recreational ST, sometimes even diagnosed as gaming disorder, which can lead to an almost complete denial of PA^56,57^, the majority of studies among healthy or representative samples report a weak relationship between PA and ST or other forms of media usage among children and adolescents^37–42^ and speak for the fact that issues among both behaviors need to be addressed separately.

## Methods

We obtained self-reported data from 1717 participants at two measurement points before (pre) and during (peri) the COVID-19 lockdown in Germany to examine the influence of the lockdown on PA and ST of children and adolescents aged 4–17 years pre. The STROBE statement^58^ guided the reporting of this study.

### Procedures

**Figure 2:**
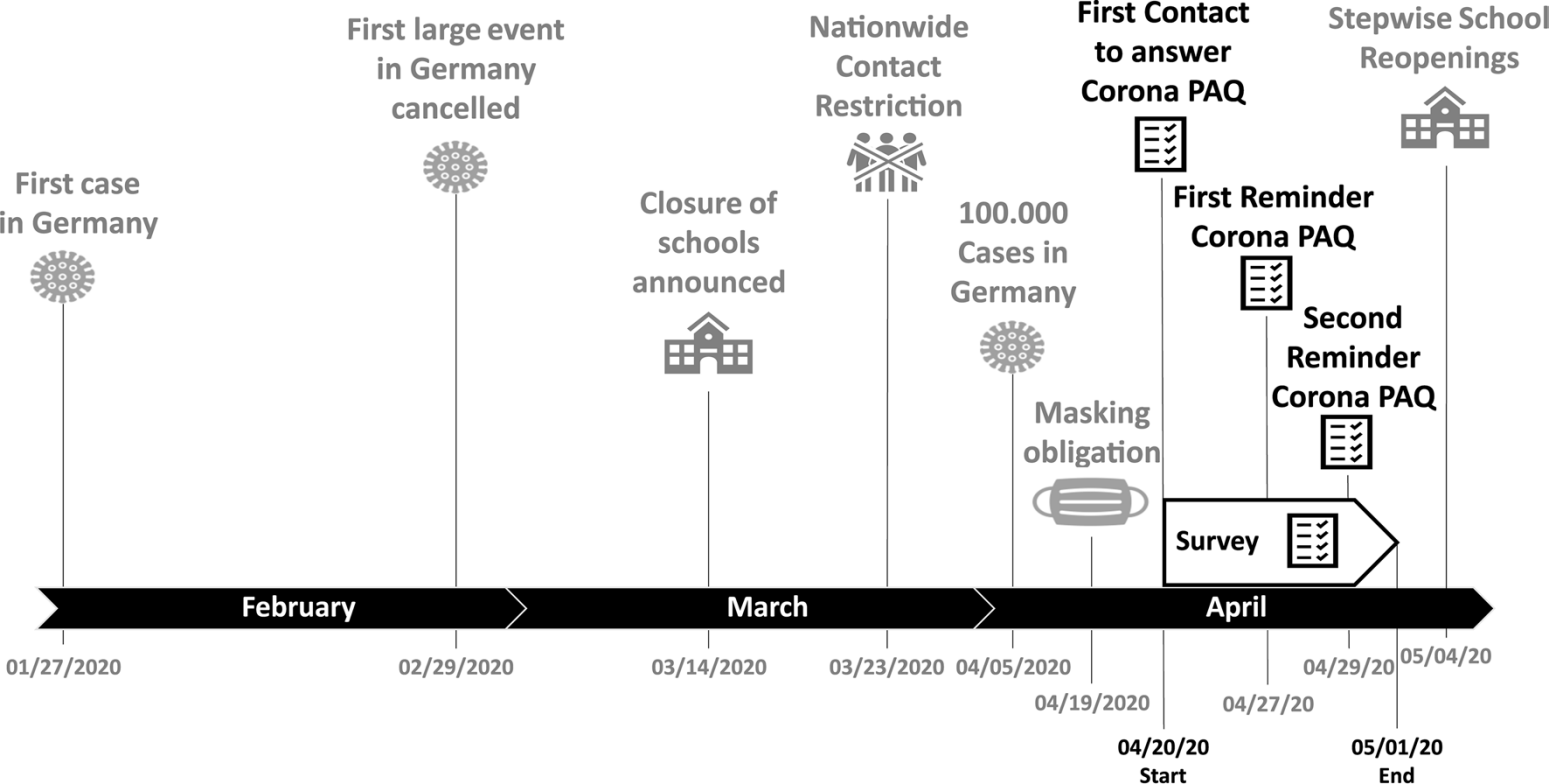
Timeline of events and survey context during the COVID-19 lockdown in Germany.

The data were obtained within the framework of the MoMo study^59^. The study follows a cohort sequence design, which means that in addition to a longitudinal observation of the MoMo participants, a new representative sample of children and adolescents living in Germany is recruited at each follow-up. The baseline data was collected from 2003 to 2006, Wave 1 was conducted between 2009 and 2012, and Wave 2 between 2015 and 2017^59^. Wave 3 started in August 2018 and was planned to be finished in June 2020, but had to be interrupted in March 2020 because of the COVID-19 lockdown in Germany. During the strictest time of the lockdown (20/04/2020–01/05/2020), we asked every participant of Wave 3 (pre-study) to answer our questionnaire again (peri-study) to track the direct changes in behavior (c.f. Fig. 2).

The study was conducted in accordance with the Declaration of Helsinki. For MoMo, ethics approval was obtained by the Charité Universitätsmedizin Berlin ethics committee, by the University of Konstanz, and the ethics committee of the Karlsruhe Institute of Technology. The Federal Commissioner for Data Protection and Freedom of Information was informed about the study and approved it.

For the pre-study, parents and children were invited to examination rooms at central locations in the proximity of their homes in the 167 cities and municipalities in which the study was conducted. Parents gave their written consent for minors and the presence of a legal guardian was mandatory under the age of 15. Questionnaires were filled out on-site by the participants on laptops (pre) or online (peri). Participation in the study was voluntary and every participant received a gift worth 20 €. The participants or their custodians were informed about the contents of the study and about data protection and gave their written consent. During the COVID lockdown in Germany, the participants were contacted via email and were asked to answer the peri-questions online.

### Participants

The MoMo participants for Wave 3, respectively the pre-study, were selected based on a nationwide multi-stage sampling approach with two evaluation levels^60^ to maximize representativeness. First, a systematic sample of 167 primary sampling units was selected from an inventory of German communities stratified according to the classification system that measures the level of urbanization and geographic distribution^61^. The probability of any community being picked was proportional to the number of inhabitants younger than 18 years of age in that community. Second, based on the official registers of local residents, an age-stratified sample of randomly selected children and adolescents was drawn. 2722 MoMo Wave 3 participants (preliminary response 25.2%) were contacted for the peri-study and data from 1711 participants were gathered. Twenty-three e-mails could not be delivered because of incorrect addresses, and a total longitudinal response of 63.6% was achieved.

Table 5 shows the characteristics of the longitudinal sample and the ages pre and peri the lockdown.

### Measures

The pre-lockdown data were derived via questionnaires (PA and ST) and interviews (socioeconomic data) on the pursuant screening site. For children under the age of 11 years, parents filled in the questionnaires together with the child. During the peri-study, the same questions about PA and ST were asked via an online version of the questionnaire except for the questions about organized PA which was shut down and forbidden by governmental law. We also changed each instance of time mentioned in the description of the questions during the peri-study to “during the Corona lockdown”.

#### Physical activity

The MoMo PA Questionnaire (MoMo-PAQ) was used to assess PA via self-reported SA and HA in different settings (sports clubs, leisure time, and school)^62^. The MoMo-PAQ consists of 28 items and measures frequency, duration, type, intensity, and setting of SA and HA. The data obtained with the MoMo-PAQ are sufficiently reliable and valid (test–retest reliability: ICC ≥ 0.68)^62^. SA at school was assessed by two items about the frequency (times per week) of 45-min classes in curricular and extracurricular sports activities which are multiplied by a factor of 8.5/12 to correct for vacations. SA in sports clubs was assessed by three items which could be answered up to four times: type of sports club activity, duration (minutes per week), and time throughout the year (months per year). Minutes were then multiplied by months per year divided by twelve to control for periodic effects of SA. Nonorganized leisure-time SA was assessed in the same way by three items which could be answered for up to four types of sports: type, duration, and time throughout the year. These items were combined in an index that reflects the daily minutes with organized (school and sports clubs) and nonorganized SA. Types of sports that do not lead to an increase in energy expenditure using large skeletal muscles (for example esports) were not defined as SA according to the definition of PA^1^, and minutes spent were multiplied with zero. Nonorganized playing outside, gardening, and housework were each assessed separately by two 8-scaled items about days per week and minutes a day in which the participants pursued the activity (“On how many days do you normally play outside*/*garden/work in the household during a week?”, “How long do you on average work/play on one of those days?”). Walking and cycling were assessed by one item each about the daily distance participants travel by walking or cycling^62^.

We also asked the participants on how many days they are active for more than 60 min with moderate to vigorous intensity to reproduce the WHO PA guideline: “Over a typical or usual week (peri: “during the Corona lockdown”), on how many days are you physically active for a total of at least 60 min per day?”^63^.

#### Recreational screen time

ST was measured via self-reported screen-time behaviors which are commonly used to report ST in youth and which have similar directions with health outcomes as direct measures of sedentary behavior^3^. Participants were asked to report the time spent watching television (TV), playing games on any device (Gaming), and using the internet for recreational use (Internet) separately for weekdays and weekends using an 7-point scale including (almost) never, 15 min per day, 30 min per day, 1 h per day, 2 h per day, 3 h per day, and 4 h per day^64^. An index reflecting weekdays and weekend days at a 5:2 ratio was then calculated for each activity.

#### Sociodemographic variables

Sociodemographic variables were assessed within the pre-study, including age, sex, and a multitude of socioenvironmental and socioeconomic variables. A detailed description of the MoMo survey instruments can be found elsewhere^65^.

### Statistics

All statistical tests were conducted using IBM SPSS 25 (IBM Corporation, Armonk, NY). Statistically significant (two-sided) differences pre and peri were determined via ANOVA for repeated measurements where the effect of time represents the lockdown effect and 95% confidence intervals for differences of units and proportions^66^, as well as effect sizes Cohen's d for pre-to-peri differences in the form of $d = \frac{{{\text{|Diff|}}}}{{{\upsigma }\left( {{\text{Diff}}} \right)}}$ were reported. Means and standard deviations were reported for PA in different settings. Means of difference and 95% CIs were reported for overall SA, HA, and ST.

## Data Availability

The datasets generated and analyzed during the current study are not publicly available due to the strict ethical standards required by the Federal Office for the Protection of Data with which study investigators are obliged to comply but are available from the corresponding author on reasonable request.

## References

[1] Caspersen CJ, Powell KE, Christenson GM (1985). Physical activity, exercise, and physical fitness: definitions and distinctions for health-related research. Public Health Rep..

[2] Janssen I, LeBlanc AG (2010). Systematic review of the health benefits of physical activity and fitness in school-aged children and youth. Int. J. Behav. Nutr. Phys. Act..

[3] Tremblay MS (2011). Systematic review of sedentary behaviour and health indicators in school-aged children and youth. Int. J. Behav. Nutr. Phys. Act..

[4] Ekelund U (2016). Does physical activity attenuate, or even eliminate, the detrimental association of sitting time with mortality? A harmonised meta-analysis of data from more than 1 million men and women. Lancet.

[5] Warburton DER, Bredin SSD (2017). Health benefits of physical activity: a systematic review of current systematic reviews. Curr. Opin. Cardiol..

[6] Poitras VJ (2016). Systematic review of the relationships between objectively measured physical activity and health indicators in school-aged children and youth. Appl. Physiol. Nutr. Metab. Physiol. Appl. Nutr. Metab..

[7] Piercy KL (2018). The physical activity guidelines for Americans. JAMA.

[8] World Health Organization. *WHO guidelines on physical activity and sedentary behaviour.* https://apps.who.int/iris/handle/10665/336656 (2020). 33369898

[9] Tremblay MS (2016). Canadian 24-hour movement guidelines for children and youth: an integration of physical activity, sedentary behaviour, and sleep. Appl. Physiol. Nutr. Metab. Physiol. Appl. Nutr. Metab..

[10] Barlow SE (2007). Expert committee recommendations regarding the prevention, assessment, and treatment of child and adolescent overweight and obesity: summary report. Pediatrics.

[11] Schmidt SCE (2020). The physical activity of children and adolescents in Germany 2003–2017: The MoMo-study. PLoS ONE.

[12] Press and Information Office of the Federal Government (2020). Agreement: Guidelines to Slow the Spread of the Coronavirus.

[13] Press and Information Office of the Federal Government (2020). Meeting of the Chancellor Angela Merkel with the Heads of Government of the German Federal States on 22 March 2020.

[14] Press and Information Office of the Federal Government (2020). Telephone Conference Between the Federal Chancellor and the Heads of Government of the Länder on 15 April 2020. Restrictions to Public Life in Order to Curb the Spread of the Covid-19 Epidemic.

[15] Brazendale K (2017). Understanding differences between summer vs. school obesogenic behaviors of children: the structured days hypothesis. Int. J. Behav. Nutr. Phys. Act..

[16] Schmidt SCE, Henn A, Albrecht C, Woll A (2017). Physical activity of german children and adolescents 2003–2012: the MoMo-study. Int. J. Environ. Res. Public Health.

[17] Rundle AG, Park Y, Herbstman JB, Kinsey EW, Wang YC (2020). COVID-19-related school closings and risk of weight gain among children. Obesity.

[18] Hall G, Laddu DR, Phillips SA, Lavie CJ, Arena R (2020). A tale of two pandemics: How will COVID-19 and global trends in physical inactivity and sedentary behavior affect one another?. Prog. Cardiovasc. Dis..

[19] Fegert JM, Vitiello B, Plener PL, Clemens V (2020). Challenges and burden of the Coronavirus 2019 (COVID-19) pandemic for child and adolescent mental health: a narrative review to highlight clinical and research needs in the acute phase and the long return to normality. Child Adolesc. Psychiatry Ment. Health.

[20] Xiang M, Zhang Z, Kuwahara K (2020). Impact of COVID-19 pandemic on children and adolescents’ lifestyle behavior larger than expected. Prog. Cardiovasc. Dis..

[21] Zenic N (2020). Levels and changes of physical activity in adolescents during the COVID-19 pandemic: contextualizing urban vs. rural living environment. Appl. Sci..

[22] Hemphill NM, Kuan MT, Harris KC (2020). Reduced physical activity during COVID-19 pandemic in children with congenital heart disease. Can. J. Cardiol..

[23] Pietrobelli A (2020). Effects of COVID-19 lockdown on lifestyle behaviors in children with obesity living in Verona, Italy: a longitudinal study. Obesity.

[24] Guerrero MD (2020). Canadian children’s and youth’s adherence to the 24-h movement guidelines during the COVID-19 pandemic: a decision tree analysis. J. Sport Health Sci..

[25] Barnes JD (2018). Results from Canada’s 2018 report card on physical activity for children and youth. J. Phys. Act. Health.

[26] Moore SA (2020). Impact of the COVID-19 virus outbreak on movement and play behaviours of Canadian children and youth: a national survey. Int. J. Behav. Nutr. Phys. Act..

[27] López-Bueno R, López-Sánchez GF, Casajús JA, Calatayud J, Gil-Salmerón A, Grabovac I (2020). Health-related behaviors among school-aged children and adolescents during the Spanish Covid-19 confinement. Front. Pediatr..

[28] Constandt B (2020). Exercising in times of lockdown: an analysis of the impact of COVID-19 on levels and patterns of exercise among adults in Belgium. Int. J. Environ. Res. Public Health.

[29] Chen P, Mao L, Nassis GP, Harmer P, Ainsworth BE, Li F (2020). Coronavirus disease (COVID-19): The need to maintain regular physical activity while taking precautions. J. Sport Health Sci..

[30] Dunton G, Do B, Wang SD (2020). Early effects of the COVID-19 pandemic on physical activity and sedentary behavior in U.S. children. Camb. Open Engage.

[31] Atkinson AB (1970). On the measurement of inequality. J. Econ. Theory.

[32] Althoff T (2017). Large-scale physical activity data reveal worldwide activity inequality. Nature.

[33] Deci EL, Ryan RM, van Lange PA (2012). Self-determination theory. Theories of Social Psychology.

[34] Bailey DP (2012). Accelerometry-assessed sedentary behaviour and physical activity levels during the segmented school day in 10–14-year-old children: the HAPPY study. Eur J. Pediatr..

[35] Justl, S. *Homeschooling in China: Between Silence and Boycott*. https://www.wuv.de/agenturen/homeschooling_in_china_zwischen_stille_und_boykott (2020).

[36] Langmeyer, A., Guglhör-Rudan, A., Naab, T., Urlen, M. & Winklhofer, U. *Childhood in Times of Corona. First Results on the Changed Everyday Life and the Well-Being of Children*. https://www.dji.de/themen/familie/kindsein-in-zeiten-von-corona-studienergebnisse.html (2020).

[37] Pearson N, Braithwaite RE, Biddle SJH, van Sluijs EMF, Atkin AJ (2014). Associations between sedentary behaviour and physical activity in children and adolescents: a meta-analysis. RObes. Rev. Off. J. Int. Assoc. Study Obes..

[38] Spengler S, Mess F, Mewes N, Mensink GBM, Woll A (2012). A cluster-analytic approach towards multidimensional health-related behaviors in adolescents: the MoMo-study. BMC Public Health.

[39] Spengler S, Mess F, Woll A (2015). Do media use and physical activity compete in adolescents? Results of the MoMo study. PLoS ONE.

[40] Feldman DE, Barnett T, Shrier I, Rossignol M, Abenhaim L (2003). Is physical activity differentially associated with different types of sedentary pursuits?. Arch. Pediatr. Adolesc. Med..

[41] Gebremariam MK (2013). Are screen-based sedentary behaviors longitudinally associated with dietary behaviors and leisure-time physical activity in the transition into adolescence?. Int. J. Behav. Nutr. Phys. Act..

[42] Melkevik O, Torsheim T, Iannotti RJ, Wold B (2010). Is spending time in screen-based sedentary behaviors associated with less physical activity: a cross national investigation. Int. J. Behav. Nutr. Phys. Act..

[43] Babic MJ (2016). Intervention to reduce recreational screen-time in adolescents: Outcomes and mediators from the ‘Switch-Off 4 Healthy Minds’ (S4HM) cluster randomized controlled trial. Prev. Med..

[44] Armstrong T, Bull F (2006). Development of the World Health Organization global physical activity questionnaire (GPAQ). J. Public Health.

[45] Garmin. *Can Fitness Find a Way? The Impact of the Global Pandemic on Human Activity: Part II*. https://bit.ly/2XmxwFF (2020).

[46] Tison GH (2020). Worldwide effect of COVID-19 on physical activity: a descriptive study. Ann. Intern. Med..

[47] Armstrong N, Welsman JR (2006). The physical activity patterns of European youth with reference to methods of assessment. Sports Med. (Auckland N.Z.).

[48] Aubert S (2018). Global Matrix 3.0 physical activity report card grades for children and youth: results and analysis from 49 countries. J. Phys. Act. Health.

[49] Ingram DK (2000). Age-related decline in physical activity: generalization to nonhumans. Med. Sci. Sports Exercise.

[50] German Weather Service. *Monthly Climate Status for Germany. April 2019*. https://www.dwd.de/DE/leistungen/pbfb_verlag_monat_klimastatus/monat_klimastatus.html (2019).

[51] German Weather Service. *Monthly Climate Status for Germany. April 2020*. https://www.dwd.de/DE/leistungen/pbfb_verlag_monat_klimastatus/monat_klimastatus.html (2020).

[52] Lewis LK (2016). At the mercy of the gods: associations between weather, physical activity, and sedentary time in children. Pediatr. Exercise Sci..

[53] Bucksch J, Inchley J, Hamrik Z, Finne E, Kolip P (2014). Trends in television time, non-gaming PC use and moderate-to-vigorous physical activity among German adolescents 2002–2010. BMC Public Health.

[54] Demetriou Y (2018). Results from Germany’s 2018 report card on physical activity for children and youth. J. Phys. Act. Health.

[55] Bucksch J (2016). International trends in adolescent screen-time behaviors from 2002 to 2010. J. Adolesc. Health Off. Publ. Soc. Adolesc. Med..

[56] Wichstrøm L, Stenseng F, Belsky J, von Soest T, Hygen BW (2019). Symptoms of internet gaming disorder in youth: predictors and comorbidity. J. Abnorm. Child Psychol..

[57] Saunders JB (2017). Gaming disorder: its delineation as an important condition for diagnosis, management, and prevention. J. Behav. Addict..

[58] Vandenbroucke JP (2007). Strengthening the reporting of observational studies in epidemiology (STROBE): explanation and elaboration. PLoS Med..

[59] Woll A, Albrecht C, Worth A (2017). Motorik-Module (MoMo)—the KiGGS wave 2 module to survey motor performance and physical activity. J. Health Monit..

[60] Kamtsiuris P, Lange M, Schaffrath Rosario A (2007). German health interview and exhamination survey for children and adolescents (KiGGS): sample design, response and nonresponse analysis. Bundesgesundheitsblatt Gesundh. Gesundh..

[61] Kurth B-M (2008). The challenge of comprehensively mapping children’s health in a nation-wide health survey: design of the German KiGGS-Study. BMC Public Health.

[62] Jekauc D, Wagner M, Kahlert D, Woll A (2013). Reliability and validity of the MoMo-physical-activity-questionnaire for adolescents (MoMo-PAQ). Diagnostica.

[63] Prochaska JJ, Sallis JF, Long B (2001). A physical activity screening measure for use with adolescents in primary care. Arch. Pediatr. Adolesc. Med..

[64] Mathers M (2009). Electronic media use and adolescent health and well-being: cross-sectional community study. Acad. Pediatr..

[65] Lampert T, Müters S, Stolzenberg H, Kroll LE (2014). Assessment of the Socio-Economic Status in the KiGGS-Study. Bundesgesundheitsblatt Gesundh. Gesundh..

[66] Cumming G, Fidler F (2009). Confidence Intervals. J. Psychol..

